# Supplementary material for: HIVprotI: an integrated web based platform for prediction and design of HIV proteins inhibitors
Source: J Cheminform. 2018 Mar 9;10:12. doi: 10.1186/s13321-018-0266-y (PMC5845081; doi:10.1186/s13321-018-0266-y)
Supplement: Supplementary file 2 — Additional file 2. Source code of HIVProtI web server. [file 13321_2018_266_MOESM2_ESM.zip › HIVprotI_Source-code/hivprotI/weka-3-6-6/doc/weka/classifiers/bayes/DMNBtext.DNBBinary.html]

DMNBtext.DNBBinary


---


|  |  |  |
| --- | --- | --- |
| **Overview** \| **Package** \| **Class** \| **Tree** \| **Deprecated** \| **Index** \| **Help** \| **Weka's home** | |  |
| **PREV CLASS**   **NEXT CLASS** | **FRAMES**    **NO FRAMES**     **All Classes** |  |
| SUMMARY: NESTED \| FIELD \| CONSTR \| METHOD | DETAIL: FIELD \| CONSTR \| METHOD |  |


---


## weka.classifiers.bayes Class DMNBtext.DNBBinary

```
java.lang.Object
  weka.classifiers.bayes.DMNBtext.DNBBinary
```

**All Implemented Interfaces:**: java.io.Serializable

**Enclosing class:**: DMNBtext

---

``` public class DMNBtext.DNBBinary extends java.lang.Object implements java.io.Serializable ```

**See Also:**: Serialized Form

---

**Constructor Summary**

`DMNBtext.DNBBinary()`


| **Method Summary** | |
| --- | --- |
| `double[]` | `distributionForInstance(Instance instance)`             Calculates the class membership probabilities for the given test instance. |
| `double` | `getLogProbForTargetClass(Instance ins)`             Calculates the class membership probabilities for the given test instance. |
| `int` | `getTargetClass()`             Gets the Target Class |
| `void` | `initClassifier(Instances instances)` |
| `void` | `setTargetClass(int targetClass)`             Sets the Target Class |
| `java.lang.String` | `toString()`             Returns a string representation of the classifier. |
| `void` | `updateClassifier(Instance ins)` |

**Methods inherited from class java.lang.Object**

`equals, getClass, hashCode, notify, notifyAll, wait, wait, wait`

**Constructor Detail**

### DMNBtext.DNBBinary

```
public DMNBtext.DNBBinary()
```


**Method Detail**

### initClassifier

```
public void initClassifier(Instances instances)
                    throws java.lang.Exception
```

:   **Throws:**: `java.lang.Exception`

---


### updateClassifier

```
public void updateClassifier(Instance ins)
                      throws java.lang.Exception
```

:   **Throws:**: `java.lang.Exception`

---


### getLogProbForTargetClass

```
public double getLogProbForTargetClass(Instance ins)
                                throws java.lang.Exception
```

:   Calculates the class membership probabilities for the given test
    instance.

    :   **Parameters:**: `instance` - the instance to be classified **Returns:**: predicted class probability distribution **Throws:**: `java.lang.Exception` - if there is a problem generating the prediction

---


### distributionForInstance

```
public double[] distributionForInstance(Instance instance)
                                 throws java.lang.Exception
```

:   Calculates the class membership probabilities for the given test
    instance.

    :   **Parameters:**: `instance` - the instance to be classified **Returns:**: predicted class probability distribution **Throws:**: `java.lang.Exception` - if there is a problem generating the prediction

---


### toString

```
public java.lang.String toString()
```

:   Returns a string representation of the classifier.

    :   **Overrides:**: `toString` in class `java.lang.Object`
    :   **Returns:**: a string representation of the classifier

---


### setTargetClass

```
public void setTargetClass(int targetClass)
```

:   Sets the Target Class

---


### getTargetClass

```
public int getTargetClass()
```

:   Gets the Target Class

    :   **Returns:**: the Target Class Index


---


|  |  |  |
| --- | --- | --- |
| **Overview** \| **Package** \| **Class** \| **Tree** \| **Deprecated** \| **Index** \| **Help** \| **Weka's home** | |  |
| **PREV CLASS**   **NEXT CLASS** | **FRAMES**    **NO FRAMES**     **All Classes** |  |
| SUMMARY: NESTED \| FIELD \| CONSTR \| METHOD | DETAIL: FIELD \| CONSTR \| METHOD |  |


---
